# Distributed Power Allocation for Wireless Sensor Network Localization: A Potential Game Approach

**DOI:** 10.3390/s18051480

**Published:** 2018-05-08

**Authors:** Mingxing Ke, Ding Li, Shiwei Tian, Yuli Zhang, Kaixiang Tong, Yuhua Xu

**Affiliations:** 1 The College of Communications Engineering, PLA Army Engineering University, Nanjing 210007, China; tianxwell@163.com (S.T.); yulipkueecs08@126.com (Y.Z.); kaixiang.tong@ucalgary.ca (K.T.); yuhuaenator@gmail.com (Y.X.); 2 Science and Technology on Communication Networks Laboratory, Shijiazhuang 050002, China; 3 Modern Educational and Technological Center, Agricultural University of South China, Guangzhou 510642, China; metclid@scau.edu.cn

**Keywords:** power allocation, localization accuracy, potential game, wireless sensor networks

## Abstract

The problem of distributed power allocation in wireless sensor network (WSN) localization systems is investigated in this paper, using the game theoretic approach. Existing research focuses on the minimization of the localization errors of individual agent nodes over all anchor nodes subject to power budgets. When the service area and the distribution of target nodes are considered, finding the optimal trade-off between localization accuracy and power consumption is a new critical task. To cope with this issue, we propose a power allocation game where each anchor node minimizes the square position error bound (SPEB) of the service area penalized by its individual power. Meanwhile, it is proven that the power allocation game is an exact potential game which has one pure Nash equilibrium (NE) at least. In addition, we also prove the existence of an ϵ-equilibrium point, which is a refinement of NE and the better response dynamic approach can reach the end solution. Analytical and simulation results demonstrate that: (i) when prior distribution information is available, the proposed strategies have better localization accuracy than the uniform strategies; (ii) when prior distribution information is unknown, the performance of the proposed strategies outperforms power management strategies based on the second-order cone program (SOCP) for particular agent nodes after obtaining the estimated distribution of agent nodes. In addition, proposed strategies also provide an instructional trade-off between power consumption and localization accuracy.

## 1. Introduction

How to acquire accurate, reliable, real-time and low-cost position information is the most pressing demand of location-based services, such as indoor positioning, asset tracking, emergency rescue, etc. [1,2,3,4,5,6,7,8,9,10]. In the outdoor scenario, the Global Navigation Satellite System (GNSS) is the most outstanding and the Global Positioning System (GPS) is the most widely-used satellite-based positioning system. However, in some harsh or indoor environments, the GNSS may not provide satisfactory localization accuracy or even not be available. This has been a motivation for research on wireless sensor network (WSN) localization in the past few decades [11].

In WSN localization systems, it is typical to employ two types of nodes: anchor nodes (infrastructure with perfect position information) and agent nodes (mobile devices with imperfect position information). Conventionally, agent nodes’ localization accuracy is depended on the network topology structure and the precision of range measurements, where the latter is related to the signal bandwidth, channel condition and transmitting power of anchor nodes [12]. At the same time, the viability of WSNs strongly depends on the power consumption of every sensor. Therefore, besides agent nodes’ localization accuracy being closely related to the power consumption of anchor nodes, the recognized lifetime also depends on it. However, higher localization accuracy is achieved at the cost of higher energy consumption, which means a reduction in the battery lifetime. Due to the energy-limited characteristic of each sensor, it is a challenging task to achieve the optimal trade-off between localization accuracy of agent nodes and power consumption of anchor nodes.

The main challenge to solve is the design of power allocation strategies. Extensive research has been done on optimization methods or cooperative localization, which play an important role in the maximization of communication and networking performance subject to total power constraints [13]. In [14], the authors considered the imperfect knowledge of network parameters and transformed the power allocation problem into semi-definite programs (SDPs) to minimize localization errors for a given power budget. In [15], the functional properties of the square position error bound (SPEB) were considered in active and passive localization networks, and the unifying optimization framework of the power allocation problem was transformed into second-order cone programs (SOCPs). To improve localization accuracy, a cooperative localization technique was proposed in [16] and an optimization framework was developed to obtain robust and efficient power allocation strategies by convex optimization approach in a cooperative WSN. As an extension of [14,15,16], the author in [17] investigated the confidence region of location inference and designed robust energy allocation strategies to minimize the position error within the confidence region.

Game theory has been widely used to develop localization algorithms in recent years [18]. Not only can the localization process [19], sleep time allocation [20], dynamic range measurement allocation [21], node selection [22] and link selection [23] be formulated as game problems, but also, the power allocation issues can be addressed as a resource management game in WSNs. In [24], the author derived a solution based on modeling the positioning problem as a supermodular game to trade-off the power level and desired positioning quality. In [25], the author proposed a power management game to obtain optimization power allocation for cooperative localization in asynchronous networks. In [18], the author formulated two power management games to minimize the SPEB of each agent under the assumptions of local and global information. Resource management issues in the power and time-frequency were solved by Stackelberg and link bargaining games in [26]. Moreover, it is imperative to highlight that a similar distributed power allocation problem was presented in our previous work [27], and the main differences are: (i) Distribution information of agent nodes and the average localization performance of service area are introduced for more practical systems in this paper. (ii) Distributed power allocation for agent nodes in cooperative localization scenarios was considered in [27], while we consider distributed power allocation for anchor nodes in this work. Also, a potential game based distributed ToA (time of arrival) localization approach was presented in [28], which is interesting and constructive.

However, the preconditions of the above studies are the restricted number and some prior information of agent nodes, such as previous positional knowledge or movement trajectories. In practice, such preconditions may not be suitable for the application of network localization techniques, and challenges still exist with the following issues.

First, in WSN localization systems, power allocation strategies should adapt to instantaneous network conditions that include not only the topology of anchor nodes, but also the distribution of agent nodes. In [14,15,16,17,18,24,25,26,27], the authors assumed that the network parameters, such as agent nodes’ positions, were perfectly known, and these parameters were obtained through estimation. In particular, for a moving agent node, the perceiving of the agent’ position in the current time slot and the predicted position in the next time slot are highly related to power allocation strategies. However, mostly the motion state of the agent node is unknown, and the strategy updates may be lagging due to the time needed for strategy calculations. Moreover, in existing power allocation algorithms, the anchor node adapts its transmitting power to satisfy every agent node’s localization requirement within its coverage. When it is considered that agent nodes are densely deployed, the anchor nodes may not afford the computational complexity. Furthermore, if the estimated position of an agent node is acquired and the localization accuracy is acceptable, it is unnecessary to update the power allocation strategies at the cost of additional operation. Therefore, the application scenarios may not be versatile.

The goal of this paper is to address the above issues from two aspects. First, the proposed method does not depend on particular agent nodes’ positions to determine the power allocation strategies of anchor nodes. While anchor nodes’ positions and the service area are mostly stationary in WSN localization systems, the power allocation strategies are determined after obtaining this information. Second, we consider the expected localization accuracy in the service area to replace the single agent node localization accuracy. Then, we resort to the prior distribution probabilities of agent nodes, rather than the motion states or the deployment of agent nodes, which may be inconsistent. Since it is critical to obtain the accurate distribution probabilities of agent nodes in advance, here we approximate them by the following methods:

(i) If we have some prior information of agent nodes, such as the central points and standard deviations of hotspots, the distribution can be modeled as a simple diffusion model [29] or Coefficient of Variation (CoV) of the Voronoi cell area [30]. It is reasonable to collect the prior information by statistical analysis or experience in some practical applications. However, all previous related works did not consider this situation, and their research may not be suitable to achieve a solution.
(ii) If we cannot obtain any prior information about agent nodes in the service area, the distribution probabilities can be estimated by anchor nodes. In this case, two phases will be implemented. In the first phase, each agent node obtains its position by unoptimized power management strategies of anchor nodes. In the next phase, anchor nodes will achieve a trade-off between power consumption and localization accuracy after estimating the distribution of agent nodes. This method may cause unavoidable estimation error compared to the actual situation. The feasibility and preliminaries are the main purpose in our work, and we will perfect this weakness.

In this paper, the power allocation problem is formulated as a power management game in a WSN localization system because game theory provides an effective analytical tool to solve distributed decision problems. Different from [24], we consider this problem from a new standpoint based on a potential game model and construct a potential function to find the end solution. The main contributions of this paper are as follows:

- We develop the service area of a WSN localization system to determine power allocation strategies. To the best of our knowledge, this is the first time that the performance of the service area, rather than particular agent nodes, has been considered.
- We propose a power management game to determine power allocation strategies of anchor nodes in a distributed WSN localization system. After designing the potential function, it is proven that the proposed game is a continuous exact potential game, and the better response algorithm can be used to find a ϵ-equilibrium point as the end solution.
- We exploit the effects of different distributions of agent nodes on the power allocation strategies when prior distribution information is available. We also derive the estimated distribution of agent nodes to manage power allocation strategies when prior knowledge is unknown. Numerical results show that different equilibrium solutions are achieved, and the performance of the proposed strategies outperforms the uniform strategies and prior power management strategies for particular agent nodes.

The rest of this paper is organized as follows. In Section 2, the system model is described, and the SPEB of the service area and the optimal objective are explained. Section 3 studies the power allocation game and presents the analysis of the equilibrium solution, which can be achieved using the better response algorithm. The simulation results are presented in Section 4. Finally, conclusions are given in Section 5.

## 2. System Models and Problem Formulation

In this section, the distributed localization system model is introduced at first. Then, the power management problem is formulated for WSN localization.

### 2.1. Network Model

**Figure 1:**
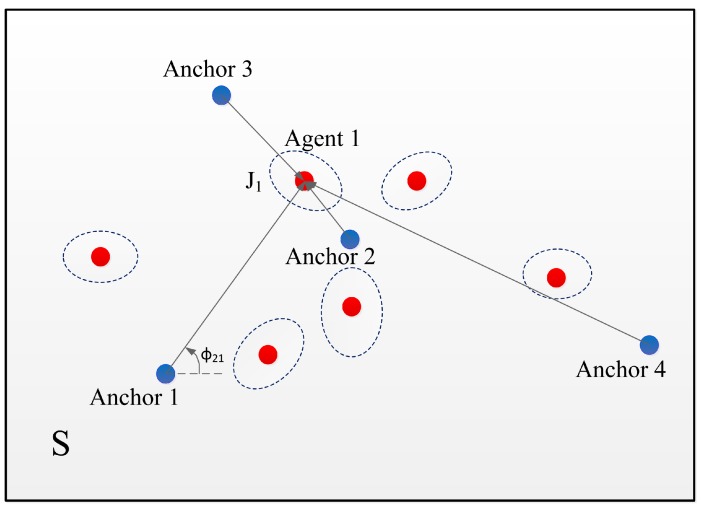
Illustration of a $N_{b}=4$ WSN localization system: anchor nodes (blue dots) communicate with agent nodes (red dots). Only the connecting relationship between Agent 1 and anchor nodes is shown. The dashed ellipses represent the location information of each agent node.

Consider a network with $N_{b}$ anchor nodes, denoted by $N_{b}=\left\{1,2,\ldots ,N_{b}\right\}$ and $N_{a}$ mobile agent nodes, denoted by $N_{a}=\left\{1,2,\ldots ,N_{a}\right\}$. Each node is deployed in a two-dimensional region, and all agent nodes are distributed in the service area S. The position of nodes *m* is denoted by $z_{m}\in R^{2}$ for $m\in N_{a}\cup N_{b}$, and the distance and angle from node *m* to anchor node *j* are denoted by $d_{mj}$ and $\phi_{mj}$, respectively. The topology of an example WSN localization system is shown in Figure 1 with 4 anchor nodes and several agent nodes which are randomly distributed in the service area S. For a particular agent node $k\in N_{a}$, let ${\hat{z}}_{m}$ be the unbiased estimation of the position $z_{m}$. Then, the mean squared error (MSE) of position estimation, denoted by $E\{{∥{\hat{z}}_{m}-z_{m}∥}^{2}\}$, can be used to quantify the localization accuracy. It has been shown in [31] that the MSE for a WSN position estimation is lower bounded by:

(1) $$E\left\{{∥{\hat{z}}_{m}-z_{m}∥}^{2}\right\}\geq \mathrm{tr}\left\{{J_{e}}^{-1}\left(z_{m};p\right)\right\},$$

where $P\left(z_{m};p\right)=\mathrm{tr}\left\{{J_{e}}^{-1}\left(z_{m};p\right)\right\}$ is the squared position error bound (SPEB) and ${J_{e}}^{-1}\left(z_{m};p\right)$ is the equivalent Fisher information matrix (EFIM) for $z_{m}$ with the power allocation vector p of anchor nodes, denoted by $p=[p_{1}\;p_{2}\;\cdots \;p_{N_{b}}]$ [14]. The EFIM for the position of agent *m* in a WSN localization system is given by [17]:

(2) $$J_{e}\left(z_{m};p\right)=\sum_{j\in N_{b}}p_{j}\xi_{mj}J_{r}\left(\phi_{mj}\right),$$

where $p_{j}$ is the transmitting power sent from anchor node *j*, $\xi_{mj}$ is the equivalent ranging coefficient (ERC) that is depended on the channel parameters, signal bandwidth and noise power [15], and $J_{r}\left(\phi \right)$ is a 2×2 matrix, called the ranging direction matrix (RDM) with angle ϕ, formulated as:

(3) $$J_{r}\left(\phi \right)\overset{\Delta }{=}\left[\begin{array}{cc} \cos^{2}\phi & \cos\phi \sin\phi \\ \cos\phi \sin\phi & \sin^{2}\phi \end{array}\right].$$

### 2.2. The SPEB of the Service Area

Since we consider power allocation strategies in a particular service area, the expected SPEB of the service area is the optimal objective, rather than the SPEB of an individual agent node. Then, the goal of localization accuracy is formulated as:

(4) $$\begin{array}{cc} \hat{P}\left(z_{\left(x,y\right)};p\right) & =\underset{(x,y)\in S}{\;\int \int \;}\mathrm{tr}\{{J_{e}}^{-1}\left(z_{\left(x,y\right)};p\right)\}f(x,y)dxdy\\ & =\underset{(x,y)\in S}{\;\int \int \;}\mathrm{tr}\left\{{\left(\sum_{j\in N_{b}}p_{j}\xi_{j}J_{r}\left(\phi_{j}\right)\right)}^{-1}\right\}f\left(x,y\right)dxdy, \end{array}$$

where $z_{\left(x,y\right)}$ is the arbitrary position of the agent node in the service area and f(x,y) is the probability density function of agent nodes distributed in the service area. $J_{r}\left(\phi_{j}\right)$ also includes variables *x* and *y*, as in the Equations (5) and (6), given by:

(5) $$\begin{array}{c} \cos\phi_{j}=\frac{\left(x-x_{j}\right)}{\sqrt{{\left(x-x_{j}\right)}^{2}+{\left(y-y_{j}\right)}^{2}}}, \end{array}$$

(6) $$\begin{array}{c} \sin\phi_{j}=\frac{\left(y-y_{j}\right)}{\sqrt{{\left(x-x_{j}\right)}^{2}+{\left(y-y_{j}\right)}^{2}}}. \end{array}$$

As can be seen in Equation (4), the solution of the double integral is intractable. Thus, we adopt the approximate estimation to transform the continuous integral into a discrete summation given by Equation (7), where *d* is the maximum diameter in the *n*-th divisory area, *N* is the total number of discrete areas, $\Delta \sigma_{n}$ is the scale of the *n*-th divisory area and $\varsigma_{n}$ and $\eta_{n}$ are arbitrary coordinate values of the *x*-axis and *y*-axis in this area.

(7) $$\begin{array}{c} \hat{P}\left(z_{\left(x,y\right)};p\right)\approx \lim_{d\to 0}\sum_{n=1}^{N}\mathrm{tr}\left\{{\left(\sum_{j\in N_{b}}p_{j}\xi_{j}J_{r}\left(\phi_{j}^{\prime }\right)\right)}^{-1}\right\}f\left(\varsigma_{n},\eta_{n}\right)\Delta \sigma_{n} \end{array}$$

Then, Equations (5) and (6) can be written as:

(8) $$\begin{array}{c} \cos\phi {}_{j}^{\prime }=\frac{\left(\varsigma_{n}-x_{j}\right)}{\sqrt{{\left(\varsigma_{n}-x_{j}\right)}^{2}+{\left(\eta_{n}-y_{j}\right)}^{2}}}, \end{array}$$

(9) $$\begin{array}{c} \sin\phi {}_{j}^{\prime }=\frac{\left(\eta_{n}-y_{j}\right)}{\sqrt{{\left(\varsigma_{n}-x_{j}\right)}^{2}+{\left(\eta_{n}-y_{j}\right)}^{2}}}. \end{array}$$

In this paper, to conveniently obtain the approximate estimation, we partition the service area though a grid division method, i.e., the scale is d×d, and $\varsigma_{n}$ and $\eta_{n}$ are chosen by the central point coordinates of the *n*-th divisory area. The error of this division method in the boundary of service areas is ignored, and the effect of different *d* values will be discussed in Section 5.

### 2.3. Problem Formulation

For an anchor node *k*, let $p_{k}$ denote the power allocation variable, and let $p_{-k}≜{\left\{p_{j}\right\}}_{j\neq k}$ denote the power allocation variable of all other anchor nodes. The SPEB of the service area is shown in (7). Then, the global network has its objective for minimizing the service area SPEB penalized by the summation of the power consumption of each anchor node [18,23], which is formulated as:

(10) $$\begin{array}{c} U\left(p_{1},\ldots ,p_{N_{b}}\right)≜\hat{P}\left(z_{(x,y)\in S};p\right)+\sum_{k=1}^{N_{b}}V_{k}\cdot p_{k}, \end{array}$$

where $V_{k}>0$ is an anchor-specific power conservation level [26] and the term $\sum_{k=1}^{N_{b}}V_{k}\cdot p_{k}$ characterizes the total power cost of all anchor nodes. The set of feasible power allocation is defined as $0⩽p_{k}⩽p_{k}^{max}$, where $p_{k}^{max}$ is the individual power constraint of anchor node *k*. Hence, the global power allocation and position estimation problem can be formulated as:

(11) $$\begin{array}{c} (P):\min U\\ \;s.t.\;0⩽p_{k}⩽p_{k}^{max},\forall k\in N_{b}. \end{array}$$

Note that there are two parts in the global network objective: the global expected localization accuracy of the service area and the summation of the power consumption of each anchor node. In other words, when an anchor node makes a decision, it not only considers itself, i.e., the power consumption $p_{k}$, but also considers the global localization performance, i.e., the expected SPEB $\hat{P}\left(z_{(x,y)\in S};p\right)$.

## 3. Potential Game for Power Allocation

From Equation (10), we see that the power allocation problem is a combinatorial optimization problem. However, to avoid a large number of calculations and communicational bottlenecks in a centralized controller [32], the transmitting power is self-determined by an anchor node according to the available myopic information. At the same time, anchor nodes interact with each other through the global optimal variable SPEB. It is a challenging problem to obtain the optimal solution with low complexity in WSNs. Such a distributed power allocation scheme in WSN localization system naturally falls into the scope of game theory and game theoretical approaches can provide an effective method to solve distributed decision problems. Thus, the former power allocation problem can be formulated as a game. Then, it can be proven that this game is a continuous exact potential game after an appropriate potential function is designed. According to some particularly good characteristics of exact potential games, there must be at least one pure strategy Nash equilibrium (NE) point, which can be considered as a steady solution. Moreover, we prove that the refinement of the NE, the ϵ-equilibrium point, can be achieved in a finite number of steps as an optimal or sub-optimal solution using the better response learning algorithm.

### 3.1. Power Allocation Game Framework

We define the power allocation game as G=(N,A,u), where $N=\{1,2,\ldots ,N_{b}\}$ represents the player set, i.e., the set of anchor nodes, A represents the strategy space set and *u* represents the utility function. The strategy profile of all players is a vector, denoted by $p=(p_{1},p_{2},\ldots ,p_{N_{b}})\in A$, and A is the joint strategy space for all players represented by $A=A_{1}⊗A_{2}⊗\ldots ⊗A_{N_{b}}$, where $A_{k}$ is the action set of player *k*, i.e., the available power $p_{k}\in \left[0,p_{k}^{max}\right]$. Let $p_{-k}=\left(p_{1},p_{2},\ldots ,p_{k-1},p_{k+1},\ldots ,p_{N_{b}}\right)\in A_{-k}$ denote the strategy profile of all players except *k* then $A_{-k}=A_{1}⊗A_{2}⊗\ldots ⊗A_{k-1}⊗A_{k+1}⊗\ldots ⊗A_{N_{b}}$. In this game, each anchor node is selfishly, and we can define the utility function of anchor node *k* as follows:

(12) $$\begin{array}{c} u_{k}\left(p_{k};p_{-k}\right)≜-\left\{\hat{P}\left(z_{(x,y)\in S};p\right)+V_{k}\cdot p_{k}\right\}. \end{array}$$

For each anchor node, it just considers its own power consumption and the global localization performance. Therefore, the proposed game can be formulated as:

(13) $$\begin{array}{c} G:\max_{p_{k}\in A_{k}}u_{k}\left(p_{k},p_{-k}\right)\;\;\forall k\in N, \end{array}$$

The NE is a straight-forward solution of the proposed game. Successively, we have the following definition of NE, which is central to a noncooperative game.

**Definition** **1** (Nash equilibrium [33]). An action profile $p^{*}=\left(p_{1}^{*},\ldots ,p_{N}^{*}\right)$ is a pure strategy Nash equilibrium (NE) if and only if no player k can improve its utility by by deviating unilaterally, i.e., if the following condition is satisfied:

(14) $$\begin{array}{c} u_{k}\left(p_{k}^{*},p_{-k}^{*}\right)⩾u_{k}\left(p_{k},p_{-k}^{*}\right),\forall k\in N,\forall p_{k}\in A_{k},p_{k}\neq p_{k}^{*}. \end{array}$$

Then, the NE is a stable solution of the game because none of player can benefit more from changing its own strategy unilaterally. Moreover, if the strategy set $A_{k}$ is a continuous interval in R and each utility function $u_{k}\left(p_{k},p_{-k}\right)$ is continuous and differentiable at the same time, G is recognized as a continuous game.

### 3.2. Analysis of NE

Firstly, the exact potential game is defined as follows.

**Definition** **2** (Exact Potential Game [33]) *The continuous game G is an exact potential game if and only if a potential function $\Phi (p_{k},p_{-k}),\forall k\in N$ satisfies the following condition:*

(15) $$\begin{array}{c} \frac{\partial \Phi (p_{k};p_{-k})}{\partial p_{k}}=\frac{\partial u_{k}\left(p_{k};p_{-k}\right)}{\partial p_{k}},\forall p_{k}\in A_{k};\forall p_{-k}\in A_{-k}. \end{array}$$

Furthermore, we have the following Lemma to imply the characteristic of proposed game.

**Lemma** **1.** *For a given power allocation model, G is an exact potential game that has at least one pure strategy NE; moreover, the optimal solution of the network power allocation problem constitutes a pure strategy NE of G.*

**Proof.**  The following proof follows the lines for proof in [28]. The potential function is formulated as follows:

(16) $$\begin{array}{c} \Phi \left(p_{k};p_{-k}\right)=-\left\{\hat{P}\left(z_{\left(x,y\right)};p\right)+\sum_{k\in N}V_{k}\cdot p_{k}\right\}. \end{array}$$

As can be seen, the potential function is the negative value of the global network optimal objective in Equation (12). Suppose that an arbitrary player *k* unilaterally changes its power allocation strategy $p_{k}$, then the change in individual utility function caused by this unilateral change is given by:

(17) $$\begin{array}{c} \frac{\partial u_{k}\left(p_{k};p_{-k}\right)}{\partial p_{k}}=-\frac{\partial \hat{P}\left(z_{\left(x,y\right)};p\right)}{\partial p_{k}}-V_{k}. \end{array}$$

Similarly, the change in the potential function caused by this unilateral change is given by:

(18) $$\begin{array}{c} \frac{\partial \Phi (p_{k};p_{-k})}{\partial p_{k}}=-\frac{\partial \hat{P}\left(z_{\left(x,y\right)};p\right)}{\partial p_{k}}-\frac{\partial (\sum_{i\in N}V_{i}\cdot p_{i})}{\partial p_{k}}. \end{array}$$

Since for player i(i≠k), its action is changeless due to the unilateral change of *k*, we have the following equation:

(19) $$\begin{array}{c} \frac{\partial (\sum_{i\in N}V_{i}\cdot p_{i})}{\partial p_{k}}=V_{k}. \end{array}$$

Hence, from Equations (17)–(19), we have:

(20) $$\begin{array}{c} \frac{\partial \Phi (p_{k};p_{-k})}{\partial p_{k}}=\frac{\partial u_{k}\left(p_{k};p_{-k}\right)}{\partial p_{k}}. \end{array}$$

Therefore, Equation (20) illustrates that the change of the individual utility function caused by the unilateral deviation of an arbitrary player equates to the change of the potential function. Thus, according to Definition 2, we can conclude that the game G is an exact potential game and Φ can be serving as the potential function.

Due to several good characteristics of the exact potential game, it has been widely used in wireless communications problems. Two of the most important characteristics are given by [33]:

(i) Every potential game has at least one pure strategy NE;
(ii) Any global or local maximum of the potential function constitutes a pure strategy NE.

Consequently, we can conclude that the global maximum of the potential function, i.e., the solution of the global optimal objective in Equation (11), is a pure positioning strategy NE point of the power allocation game G. What is more, the potential function may have a local maximum corresponding to another sub-optimal NE point. Thus, Lemma 1 is proven. ☐

### 3.3. Achieving the ϵ-Equilibrium

We have proved the existence of NE in proposed game, then how to identify and find it is the following work. In potential games, several approaches are proposed to get a pure NE, e.g., the spatial adaptive play (SAP) [34], fictitious play (FP) [35], best response [28,36], better response [27,37], etc. The ultimate purpose of such decision rules is to drive the game towards an NE based on only available myopic information. Among the methods, there are two most important and commonly-used decision rules, namely, the best response dynamics and better response dynamics [33].

As it is very complicated to find the best response strategy at each iteration in the proposed game, we can use the better response learning algorithm to find the end solution in this paper. Note that it is discrete to achieve the end solution by the better response learning algorithm, but it be proven that the difference between the end solution and the actual NE is an arbitrary small error and the number of iteration is finite. At the same time, the computational complexity is lower than that of other algorithms [33].

First, the pure strategy $p_{i}$ of player *i* is extended to a mixed strategy, denoted by $p_{i}\left(t\right)$ at iteration *t*. Moreover, to guarantee the convergence in finite steps, the ϵ-improvement path can be used, and we have the following definition.

**Definition** **3** (ϵ-improvement path [33]). *A path ρ=[p(0),p(1),…,p(t),…] is an ϵ-improvement path if in each step t, the deviating player k experiences $u_{k}\left[p\left(t\right)\right]>u_{k}\left[p\left(t-1\right)\right]+\epsilon$, for some $\epsilon \in R_{+}$.*

In addition, this facilitates the ϵ-equilibrium, which is a profile that is approximately close to an actual NE.

**Definition** **4** (ϵ-equilibrium [33]). The strategy profile $p^{*}\in A$ is an ϵ-equilibrium if and only if ∃ϵ∈R+, such that, ∀k∈N:

(21) $$\begin{array}{c} u_{k}\left(p_{k}^{*},p_{-k}^{*}\right)⩾u_{k}\left(p_{k},p_{-k}^{*}\right)-\epsilon ,\forall p_{k}\in A_{k}. \end{array}$$

The ϵ-equilibrium can be recognized as a refinement of the original NE, even in some cases, it is preferred as a solution that requires lower computational complexity.

**Proposition** **1.** *For the continuous exact potential game G with bounded utility function $u_{k}$ in Equation (12), every ϵ-improvement path is finite. The end of this path is an ϵ-equilibrium, which is a refinement of NE in Equation (13).*

**Proof.**  Note that the following steps are inspired by the similar proof given in [33]. In the service area S, the EFIM is finite and non-zero in (2). Then, the SPEB of the service area is bounded with limited power allocation scopes. Therefore, the utility function $u_{k}$ is bounded, and the potential function must be bounded, too. So, ∃L∈R,L<∞ such that L=supΦ(p),p∈A.

Now, suppose that ϱ=[p(0),p(1),p(2),…,p(t),…] is an ϵ-improvement path, which is infinite. By definition,

(22) $$\begin{array}{c} u_{k}\left[p\left(t\right)\right]-u_{k}\left[p\left(t-1\right)\right]>\epsilon ,\forall k. \end{array}$$

Because G is an exact potential game,a sufficiently small constant $\epsilon^{\prime }$ is existed and it satisfies:

(23) $$\begin{array}{c} \Phi \left[p\left(t\right)\right]-\Phi \left[p\left(t-1\right)\right]>\epsilon^{\prime },\forall k. \end{array}$$

This implies:

(24) $$\begin{array}{c} \Phi \left[p\left(t\right)\right]-\Phi \left[p\left(0\right)\right]>t\epsilon^{\prime }, \end{array}$$

or:

(25) $$\begin{array}{c} \Phi \left[p\left(t\right)\right]>\Phi \left[p\left(0\right)\right]+t\epsilon^{\prime },\forall k. \end{array}$$

Clearly, $\lim_{t\to \infty }\Phi \left[p\left(t\right)\right]=\infty$, which is a contradiction.

Therefore, for an arbitrary ϵ-improvement path, it must terminate after certain *T* steps, where $\Phi \left[p\left(t\right)\right]\leq L<\Phi \left[p\left(t\right)\right]+\epsilon^{\prime }$ or $\Phi \left[p\left(t\right)\right]>L-\epsilon^{\prime }$. It means that an ϵ-equilibrium can be reached at the end point. In other words, for the proposed game G with the bounded utility function in Equation (12), any ϵ-improvement path will converge to an ϵ-equilibrium point in finite steps. What is more, the ϵ-equilibrium can be recognized as a refinement of the original NE [33]. Therefore, Proposition 1 is proven. ☐

Using the theoretical result above and inspiring by [27], an efficient power management algorithm under the power allocation game G is given in Algorithm 1.   

**Algorithm 1** Better response-based power allocation algorithm.
**Step** **(1)**Initial stage: set *t*=1, the initial power allocation strategy of each anchor node is randomly selected from its power scope; set positions of each anchor nodes and the service area S; set the power adjusted step $\Delta p_{k}\left(1\right)=p_{k}^{\max}/4$; set the ϵ-equilibrium threshold ϵ.**Step** **(2)**Every anchor node exchanges information with the others.**Step** **(3)**The anchor node is selected in a round-robin manner, say *k*.**Step** **(4)**All the others repeat their strategies, i.e., $p_{-k}\left(t+1\right)=p_{-k}\left(t\right)$. At the same time, anchor node *k* can calculate its utility function with the information received from the other anchor nodes through (10), i.e., $u_{k}\left(p_{k},p_{-k}|t\right)$.**Step** **(5)**Anchor node *k* changes its strategy by $p_{k}^{{}^{\prime }}\left(t\right)=p_{k}\left(t\right)+\Delta p_{k}\left(t\right)$ and $p_{k}^{{}^{\prime \prime }}\left(t\right)=p_{k}\left(t\right)-\Delta p_{k}\left(t\right)$ to obtain the utility function $u_{k}^{{}^{\prime }}\left(p_{k}^{{}^{\prime }},p_{-k}|t\right)$ and $u_{k}^{{}^{\prime \prime }}\left(p_{k}^{{}^{\prime \prime }},p_{-k}|t\right)$. If all the strategies are in the power scope, let $A_{k}^{*}\left(t\right)=\left[p_{k}\left(t\right),p_{k}^{{}^{\prime }}\left(t\right),p_{k}^{{}^{\prime \prime \prime \prime }}\left(t\right)\right]$, else delete the incongruent element in this set. Then, the next strategy is selected to maximize the utility function in $A_{k}^{*}\left(t\right)$, which satisfies:
(26)$$\begin{array}{c} p_{k}\left(t+1\right)=\arg\max_{p_{k}\in A_{k}^{*}}u_{k}\left(p_{k},p_{-k}|t\right). \end{array}$$
If $p_{k}\left(t+1\right)=p_{k}\left(t\right)$, then $\Delta p_{k}\left(t+1\right)=\Delta p_{k}\left(t\right)/2$; else $\Delta p_{k}\left(t+1\right)=\Delta p_{k}\left(t\right)$.**Step** **(6)**When $p_{k}\left(t+1\right)\neq p_{k}\left(t\right)$ and $|u_{k}\left[p_{k}\left(t+1\right),p_{-k}\left(t+1\right)\right]-u_{k}\left[p_{k}\left(t\right),p_{-k}\left(t\right)\right]|⩽\epsilon$ are satisfied, player *k* will keep its strategy invariable in following iterations.**Step** **(7)**If reaching the ϵ-equilibrium points, stop; else, if the round-robin selection finishes, t=t+1, and go to Step 2; else, go to Step 3.


Note that in Step 2, the exchanged information between nodes is the current power state $p_{k}\left(t\right)$ of each anchor node; on the other hand, the position of each node is also one part of the exchanged information the first time, then it will be invariable as the anchor nodes are static. In this paper, we consider a fully-connected network, where anchor nodes can communicate with each other.

Note that in Step 5, the change step is designed as plus or minus $\Delta p_{k}\left(t\right)$, which is used to find the improved path. The value of $\Delta p_{k}\left(t\right)$ will decrease the algorithm convergence rate if it is too small or large, so we set its initial value as $p_{k}^{\max}/4$, and it will decrease by half with the strategies’ dynamics. Though the selection strategy is the best one in the sun-set $A_{k}^{*}\left(t\right)$ in Equation (26), it is still a better strategy in the whole strategy space set.

**Lemma** **2.** *For the continuous exact potential game G, the better response learning algorithm can guarantee that the end solution converges to an ϵ-equilibrium, which is the optimal or sub-optimal solution of the optimal objective in Equation (11).*

**Proof.**  Firstly, we give the definition of better response dynamics [33]: in better response dynamics, player *k* will choose a new strategy $p_{k}\left(t+1\right)$ over the current strategy $p_{k}\left(t\right)$ if and only if the new strategy $p_{k}\left(t+1\right)$ can improve its payoff, given the opponents’ strategy $p_{-k}\left(t\right)$, which can be expressed by (27).

(27) $$\begin{array}{c} p_{k}\left(t+1\right)=\left\{p_{k}^{\prime }\left(t\right)|p_{k}^{\prime }\left(t\right)\in A_{k},u_{k}\left[p_{k}^{\prime }\left(t\right),p_{-k}\left(t\right)\right]>u_{k}\left[p_{k}\left(t\right),p_{-k}\left(t\right)\right]\right\},\forall k\in N. \end{array}$$

Therefore, in the continuous exact potential games G, for ∀k∈N, there is $\exists \epsilon_{0}\in R_{+}$, which fits:

(28) $$\begin{array}{c} u_{k}\left[p\left(t+1\right)\right]>u_{k}\left[p\left(t\right)\right]+\epsilon_{0}. \end{array}$$

This means the better response learning algorithm provides an ϵ-improvement path given in Definition 3. Then, combined with Proposition 1, it can be guaranteed that the end point of the ϵ-improvement path converges to an ϵ-equilibrium through the better response learning algorithm. Therefore, Lemma 2 is proven. A more detailed derivation is presented in Section 2.2 of [33].

According to Proposition 1 and Lemma 2, the proposed algorithm can advance towards an ϵ-equilibrium in finite iterations, which can be recognized as the optimal or sub-optimal solution of the optimal objective because it is approximately close to an actual NE.

### 3.4. Complexity Analysis

The computational complexity of proposed algorithm can be evaluated through calculating the number of operations involved [24]. Table 1 shows a total number of operations in one iteration. The complex operations mainly focus on determining the SPEB of the service area. When considering the upper bound in No. 7, the computational cost for an anchor node *i* in one iteration can be given by:

(29) $$\begin{array}{c} C_{i}\leq O\left(27NN_{b}+51N-1\right). \end{array}$$

Note from Equation (29) that the computational complexity scales with *N*, which is the total number of discrete areas in the service area, and we can approximate it by $N\approx S/d^{2}$ where *S* is the size of the service area. On the other hand, when the convergence time $N_{it}$ is taken into account, the computational cost for an anchor node *i* can be expressed by:

(30) $$\begin{array}{c} C_{i}^{\prime }\leq O\left[N_{it}\left(\left(27N_{b}+51\right)\frac{S}{d^{2}}-1\right)\right]. \end{array}$$

This is consistent with the common sense that the computational cost is positively correlated to the number of anchor nodes, the size of the service area and the convergence time, and it is inversely related to the scale of the partition. The convergence time is related to the ϵ-equilibrium threshold, so the ϵ should not be set too small to avoid a large computational cost. Moreover, when considering the value of the partition step size *d*, it should have an appropriate trade-off between the computational complexity and the approximation error.

## 4. Numerical Results

In this section, the performance of the game-theoretic power allocation algorithm is evaluated. The simulation setup is first presented. Then, the simulation results are described from two aspects: with prior distribution information and without prior distribution information of agent nodes.

### 4.1. Scenario Setup

**Figure 2:**
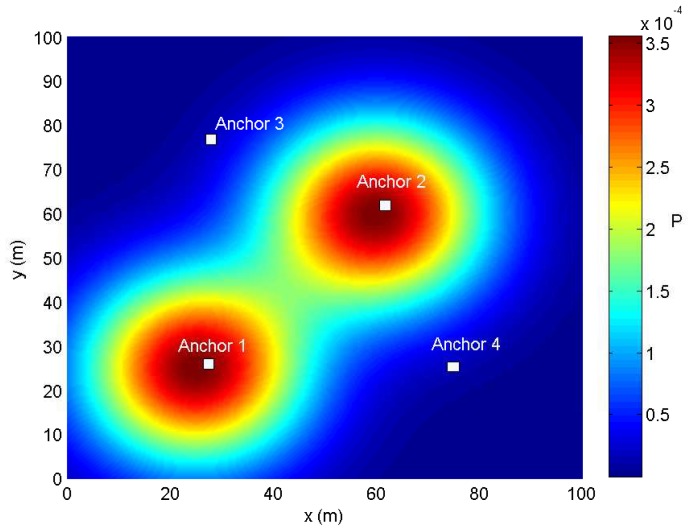
The WSNs consists of four anchor nodes, and the service area is the 100 m × 100 m square region and two special focus areas with a higher value of the PDF. The different colors represent different probabilities as indicated on the right.

We consider the scenario shown in Figure 2. In this scenario, the service area is a 100 m × 100 m region, and a set of $N_{b}$ = 4 anchor nodes is distributed at regular known positions. The ranging signals are considered with carrier frequency $f_{c}=2.1$ GHz and bandwidth W=40 MHz. The noise power density is −168 dBm/Hz. The ranging signal propagation model adopts the WINNERchannel model [38] as follows:

(31) $$Pathloss\left[dB\right]=A+B\log_{10}d\left[m\right]+20\log_{10}\frac{f_{c}\left[GHz\right]}{5.0}+X,$$

where $X∼N(0,\sigma^{2})$ accounts for the shadow fading. For anchor node transmission, the parameters are set as *A* = 41.0, *B* = 23.8 and σ=4 [16]. Moreover, we can obtain the ERCs ξ according to the formulas in [31]. For each anchor node, the maximum power $p^{max}$ is set as 1 Watt. For a small threshold ϵ, the convergence time is longer, but the ϵ-equilibrium is closer to an actual NE, and vice versa. Here, the ϵ-equilibrium judgment is set as $10^{-4}$ for a trade-off between convergence time and estimated error.

### 4.2. Performance with Prior Knowledge

We first consider a scenario where the prior distribution information of agent nodes is known. Such assumption is a reasonable approximation according to some realistic scenarios, especially in some auditoriums or storehouses where agent nodes are distribute within some special area and mostly keep static. The prior information can be collected by statistical analysis or experience in some practical applications. Then, the proposed algorithm is evaluated in three cases:

- Case 1: All the agent nodes are uniformly distributed in the service area, which means the probability density function is f(x,y)=1/S.
- Case 2: In a general way, agent nodes may focus on some special areas such as a passageway or a seat in an auditorium, called hotspots. However, the outline of special areas and the probability density should be set according to the actual condition. In this paper, the distribution of agent nodes is simply set analogous to the nodes’ placement in sensor networks, which is called as a simple diffusion model in [29]. Then, we consider that there are two special areas as shown in Figure 2, and the probability density function (PDF) of agent nodes’ positions is given by:
  (32) $$\begin{array}{cc} f^{\prime }\left(x,y\right) & =\{\frac{1}{2\pi \sigma_{1}\sigma_{2}}exp\left\{-\frac{1}{2}\left[\frac{{\left(x-25\right)}^{2}}{\sigma_{1}^{2}}+\frac{{\left(y-25\right)}^{2}}{\sigma_{2}^{2}}\right]\right\}\\ & +\frac{1}{2\pi \sigma_{3}\sigma_{4}}exp\left\{-\frac{1}{2}\left[\frac{{\left(x-60\right)}^{2}}{\sigma_{3}^{2}}+\frac{{\left(y-60\right)}^{2}}{\sigma_{4}^{2}}\right]\right\}\}/. \end{array}$$
  Here, we set the central point of special areas as (25,25) and (60,60), which are close to two anchor nodes, and the variances are $\sigma_{1}=\sigma_{2}=\sigma_{3}=\sigma_{4}=15$. The is divided by the normalization of the PDF in the service area given by:
  (33) $$\begin{array}{cc} \Delta & =\underset{(x,y)\in S}{\;\int \int \;}\{\frac{1}{2\pi \sigma_{1}\sigma_{2}}exp\left\{-\frac{1}{2}\left[\frac{{\left(x-25\right)}^{2}}{\sigma_{1}^{2}}+\frac{{\left(y-25\right)}^{2}}{\sigma_{2}^{2}}\right]\right\}\\ & +\frac{1}{2\pi \sigma_{3}\sigma_{4}}exp\left\{-\frac{1}{2}\left[\frac{{\left(x-60\right)}^{2}}{\sigma_{3}^{2}}+\frac{{\left(y-60\right)}^{2}}{\sigma_{4}^{2}}\right]\right\}\}dxdy. \end{array}$$
- Case 3: For more general situations, agent nodes are non-uniformly distributed as a simple diffusion model, but the central point of the special area is a random distribution in the service area. Different from Case 2, we assume that the central point is $c(x_{c},y_{c})$, and the PDF of agent nodes’ positions is:
  (34) $$\begin{array}{c} f^{\prime \prime }\left(x,y\right)=\left\{\frac{1}{\pi \sigma_{5}\sigma_{6}}exp\left\{-\frac{1}{2}\left[\frac{{\left(x-x_{c}\right)}^{2}}{\sigma_{5}^{2}}+\frac{{\left(y-y_{c}\right)}^{2}}{\sigma_{6}^{2}}\right]\right\}/\Delta \prime \right\}, \end{array}$$
  where the variances are $\sigma_{5}=\sigma_{6}=15$ and *c* is a two-dimensional uniform distribution in the service area S. The Δ′ is also used to normalize the PDF defined by:
  (35) $$\begin{array}{c} \Delta \prime =\underset{(x,y)\in S}{\;\int \int \;}\frac{1}{\pi \sigma_{5}\sigma_{6}}exp\left\{-\frac{1}{2}\left[\frac{{\left(x-x_{c}\right)}^{2}}{\sigma_{5}^{2}}+\frac{{\left(y-y_{c}\right)}^{2}}{\sigma_{6}^{2}}\right]\right\}dxdy. \end{array}$$

The performance of the proposed algorithm is compared with another two methods:

- Max-power: Each anchor node transmits the maximum power, i.e., $p_{k}=p_{k}^{max}$. This is the practical situation without power optimization;
- The proposed game power allocation: Every anchor node obtains the power allocation strategy through Algorithm 1, i.e., $p_{k}=p_{k}^{game}$;
- Equal-power: All anchor nodes adopt the uniform power allocation strategies with the same total power in the proposed game power allocation strategies [16], i.e., $p_{k}=\left(\sum_{k=1}^{N}p_{k}^{game}\right)/N$.

To illustrate the performance of the proposed algorithm, we find the best power allocation strategies through an exhaustive search method in different cases.

#### 4.2.1. Power Allocation Strategies

**Figure 3:**
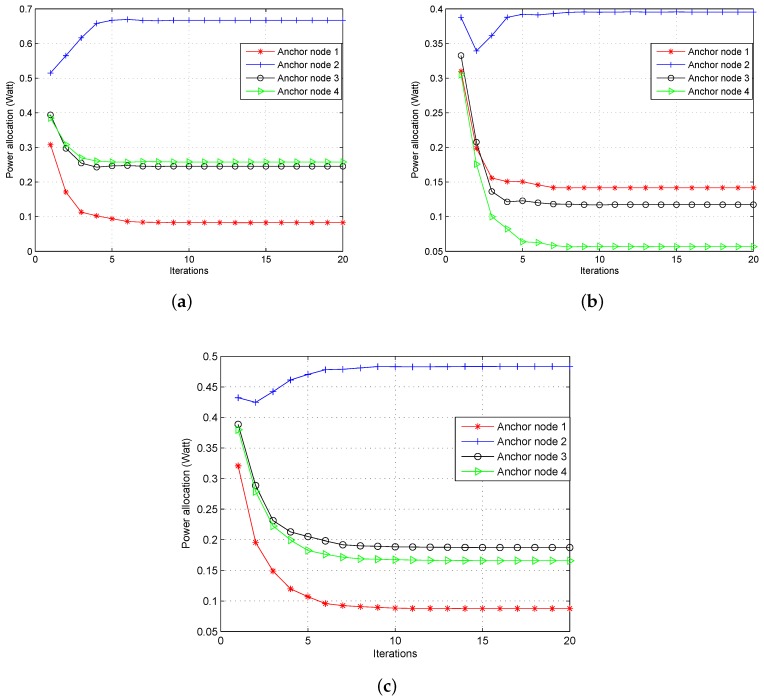
Convergence for power allocation in different cases. (**a**) Case 1; (**b**) Case 2; (**c**) Case 3.

First, we investigate the transmitting power of each anchor node change with iterations. Figure 3 shows the transmitting power at each iteration of the game in the three cases, where $V_{k}$ = 0.2 and d=2 m. The results of the proposed algorithm were averaged over 100 independent Monte Carlo trials to illustrate performance. It can be seen that the power of each anchor node is updated before reaching the equilibrium point. Note that the anchor node in the central place is allocated more power than the nodes in the marginal area, such as Agent Node 2 in this scenario. This is consistent with common sense that the node in the central place makes more of a contribution to the localization of the service area than in the marginal area. When the agent nodes are non-uniformly distributed as in Case 2 and Case 3, the equilibrium point of each anchor node is changed and shown in Figure 3b,c, respectively. Compared to Case 1, the power of Node 1 increases from 0.082 Watt to 0.142 Watt, while the others all decrease in Case 2. This is due to the particular PDF of agent nodes in the service area, In Case 3, it can be seen that the power of each anchor decreases, except for Node 1. Moreover, the total power consumption is 1.253 Watt, 0.711 Watt and 0.925 Watt in these three cases, respectively. Therefore, when the distribution of agent nodes is considered, the power consumption of the WSN localization system is decreased by more than 43% in Case 2 and 26% in Case 3 compared to the uniform distribution in Case 1.

#### 4.2.2. The SPEB in Different Power Allocation Strategies

**Figure 4:**
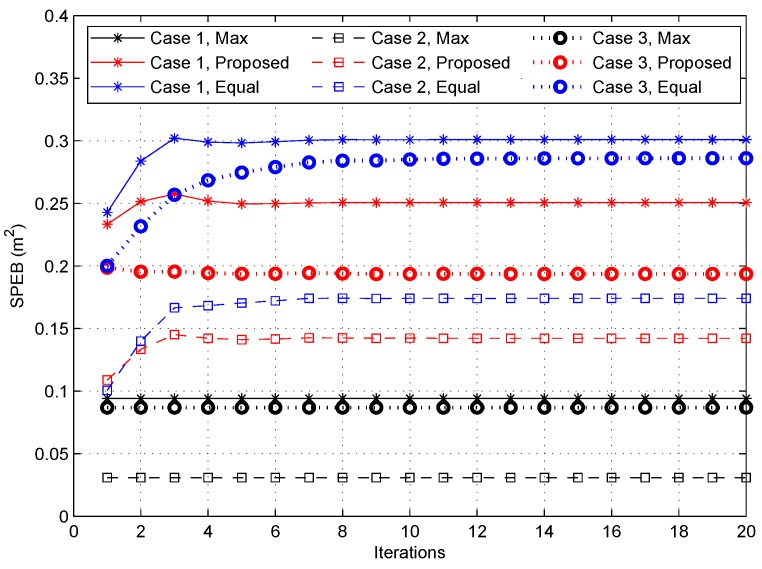
The square position error bound (SPEB) for different strategies in three cases.

Figure 4 illustrates the average SPEB with respect to iterations for all strategies in three cases. First, consistent with intuition, the max-power allocation strategy can reach lowest SPEB in each case. However, the cost is the highest power consumption, 4 Watt, compared with other strategies consuming 1.253 Watt, 0.711 Watt and 0.925 Watt at their equilibrium points in Case 1 to Case 3, respectively. Second, the proposed power allocation strategy performs better than the uniform strategy, reducing the SPEB by more than 16%, 18% and 32% in Cases 1, 2 and 3. Third, the SPEB in Case 2 outperforms Case 1 and Case 3, reducing the SPEB by more than 43% and 26%, respectively, for the proposed power allocation strategy. This can be attributed to the fact that more information about the distribution of agent nodes enhances the localization performance.

#### 4.2.3. Localization Performance with Respect to Partition Step Size *d*

**Figure 5:**
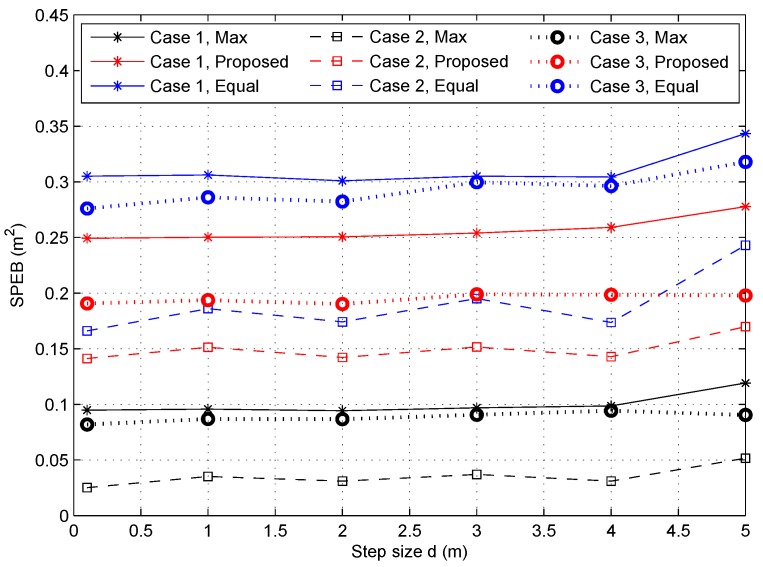
The SPEB of service area with respect to different partition step sizes *d*.

Due to the approximate estimation used to transform the continuous integral into a discrete summation as shown in Equation (7), it is a critical problem to determine the partition step size *d*. If the step size is too large, the computational complexity is low, but the results will be different from the real situation, and vice versa. Figure 5 shows the SPEB of the service area for different partition step sizes from 0.1 m to 5 m when $V_{k}$ = 0.2. As we can see in Figure 5, in Case 1, the SPEB of the service area is almost the same for each strategy when d⩽3m. With the *d* increasing, the SPEB clearly increases due to the approximation error. In Case 2, the fluctuation of the SPEB is clear. This is because borders of different probability density areas affect the solution in different partition schemes. However, in Case 3, changes in the SPEB are not obvious for different step sizes *d* due to the random distribution of the central point in the PDF. Note that the gaps of Case 1 and Case 3 in max-power or equal-power strategies are small; this is because the results are averaged over 100 independent Monte Carlo trials. Due to the trade-off between localization performance and computational complexity, we choose d=2 m in this paper, as it seems reasonable for the approximation of the continuous integral solution.

#### 4.2.4. Performance of Different Anchor-Specific Power Conservation Levels $V_{k}$

**Figure 6:**
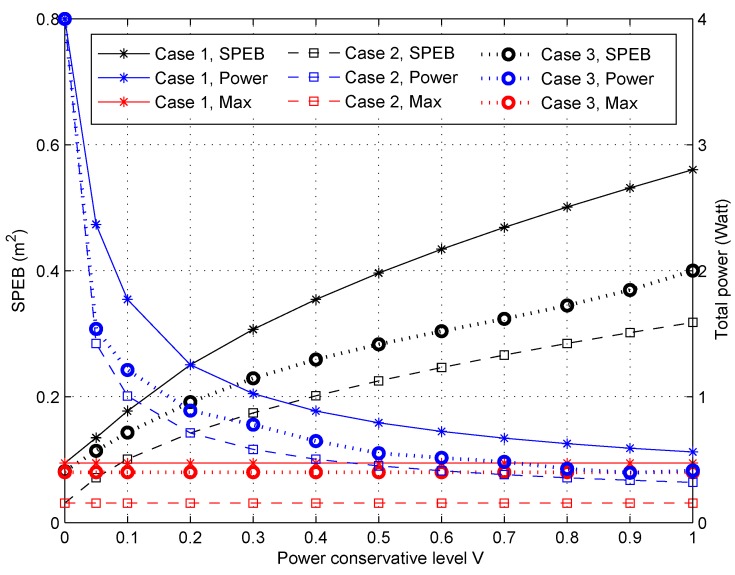
The SPEB of service area and total power consumption of anchor nodes with respect to different anchor-specific power conservation levels *V*.

We also compare the performance of the proposed algorithm for different power conservation levels $V_{k}$. Note that each anchor node can choose its specific power conservation level $V_{k}$, but the same value *V* is considered for each anchor node to facilitate comparison. The SPEB of the service area and the total transmitting power with respect to different *V* are shown in Figure 6. The left vertical coordinate correspond to the SPEB, while the total power is shown on the right side in Figure 6. First, it can be seen that the SPEB of the service area increases with *V*, in contrast the total power decreases in both cases. Besides, if *V* is close to zero, the SPEB is close to the result of max-power allocation strategy. This means that the system cares about localization accuracy more than power consumption. For large *V*, the total transmitting power is lower, but the localization accuracy is worse with a higher SPEB. We can conclude that the power conservation level *V* establishes a trade-off between localization accuracy and power consumption. Finally, the performance in Case 2 is better than in Case 1 and Case 3. This is because we consider a practical distribution of agent nodes to optimize power allocation strategies, where agent nodes are concentrated around some anchor nodes. For the general situation in Case 3, the performance is better than in Case 1. The lower power consumption and the lower SPEB show the outperformance of the proposed algorithm when considering the distribution of agent nodes.

**Figure 7:**
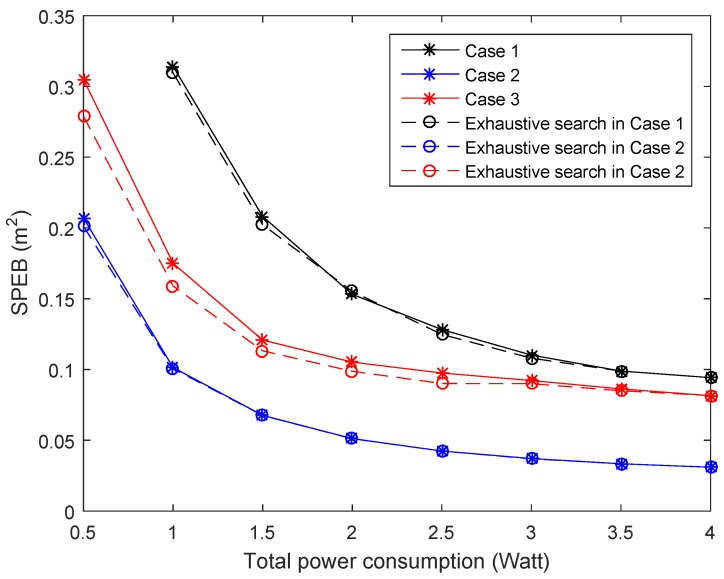
The SPEB of service area with respect to the total power consumption by proposed algorithm and the exhaustive search method in different cases.

To clearly illustrate the performance of Algorithm 1, Figure 7 demonstrates the expected SPEB of the service area with respect to the total power consumption by different methods. For exhaustive search results, the power of each anchor node is transformed into a discrete value with the least scale of 0.001 Watt. Although the power allocation strategy of the exhaustive search achieves slightly lower performance in Case 3, there is almost identical performance in Case 1 and Case 2. It is shown that the proposed algorithm can converge closely to the global optimal solution in such cases. Meanwhile, the complexity for proposed algorithm is much less than the exhaustive search method, which is related to the strategy space set and increases exponentially with the anchor nodes’ number. Because of the stochastic behavior in Case 3, the proposed algorithm may achieve the sub-optimal solution of the optimal objective. Therefore, here exists a gap between the proposed algorithm and exhaustive search.

### 4.3. Performance without Prior Knowledge

We then consider scenarios in which prior knowledge is unavailable. In such cases, the distribution information of agent nodes is unknown. This situation is also common, such as some outdoor squares where agent nodes are distributed irregularly and dynamically.

Here, the power allocation strategies can be solved by the following two phases. In the first phase, all agent nodes get their positions by the maximum transmitting power of each anchor node. Then, according to the obtained positions, the distribution probability of agent nodes can be estimated by anchor nodes. In our work, we simplify the uncertainty model of agent nodes’ estimated positions and ignore the effect of localization error on the distribution probability. In the next phase, the power allocation strategies can be reached as the same method used in Section 4.2. Since the same method is used, similar simulation results are not repeated here. To evaluate the proposed algorithm, we adopt two cases:

- Case 4: There are 20 agent nodes distributed around the hotspot point (50,50);
- Case 5: There are 100 agent nodes distributed around the hotspot point (50,50).

**Figure 8:**
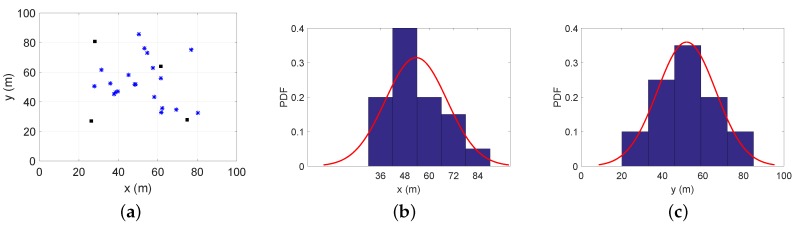
Estimation for 20 agent nodes’ distribution: (**a**) actual positions; (**b**) the estimation for the *x*-axis distribution probability; (**c**) the estimation for the *y*-axis distribution probability. The red curves are the estimated distribution probabilities, and the histograms are the actual distribution probabilities.

**Figure 9:**
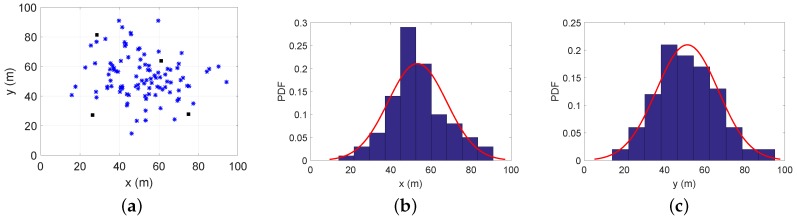
Estimation for 100 agent nodes’ distribution: (**a**) actual positions; (**b**) the estimation of the *x*-axis distribution probability; (**c**) the estimation of *y*-axis the distribution probability.

To gain some insights, Figure 8 and Figure 9 demonstrate estimations for Case 4 and Case 5. After obtaining the positions of agent nodes, we can calculate the probability density function about the *x*-axis and *y*-axis in the service area as the histograms in Figure 8b,c and Figure 9b,c. The distribution probability of agent nodes in the service area can be calculated through fitting methods shown as the red curves. Combining subfigures (b) and (c) in Figure 8 or Figure 9, we can get the PDF of the agent nodes’ positions in the two-dimensional service area for different cases.

Then, different power allocation strategies is also compared in the same scenario:

- Second-order cone program (SOCP): The power allocation problem was transformed into second-order cone programs in [15]. While the strategies only considered particular agent nodes, due to multiple agent nodes being distributed in the service area, we get the power allocation strategy for every agent node and the average SPEB for all agent nodes at the same time. For example, for the particular Agent Node 1, anchor nodes will obtain a power allocation strategy, and the average SPEB for all agent nodes can be calculated with this strategy. Then, we choose the strategy with the least average SPEB to compare with proposed strategies.
- The game strategies for particular agents: When only a particular agent is considered, the power allocation strategies also can be obtained by a potential game approach. Different from the proposed approach, the optimal objective is given by:
  (36) $$\begin{array}{c} \left(P^{\prime }\right):\min U^{\prime }≜\min\left[P\left(z_{m};p\right)+\sum_{k=1}^{N_{b}}V_{k}\cdot p_{k}\right],\\ \;s.t.\;\;0⩽p_{k}⩽p_{k}^{max},\forall k\in N_{b},\forall m\in N_{a}. \end{array}$$
  With the similar proof method and solving algorithm of the proposed approach, we can get the power allocation strategies for $\forall m\in N_{a}$. Here, we also choose the strategy with the least average SPEB to compare with different strategies.
- The proposed strategy for the estimation distribution: When the distribution of agent nodes is estimated, each anchor node obtains the power allocation strategy through the proposed Algorithm 1.

#### Performance of Different Strategies

**Figure 10:**
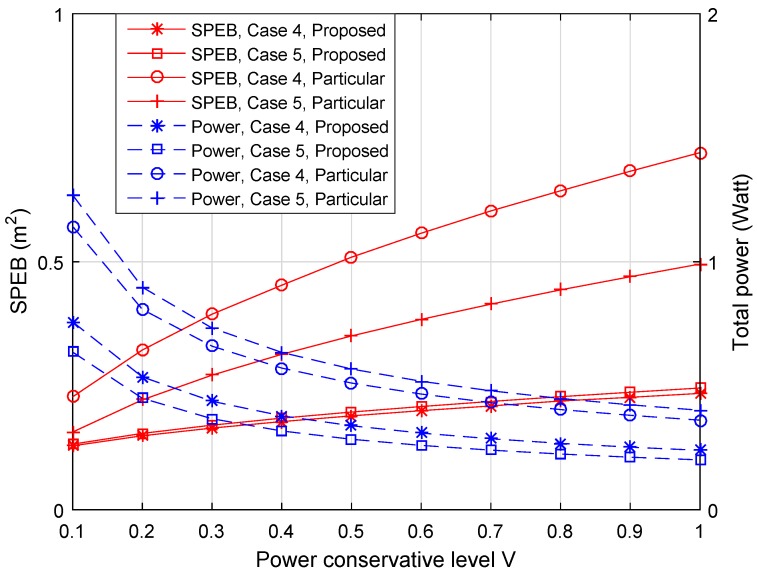
The average SPEB of agent nodes and total power consumption of anchor nodes with respect to different anchor-specific power conservation levels *V*.

**Figure 11:**
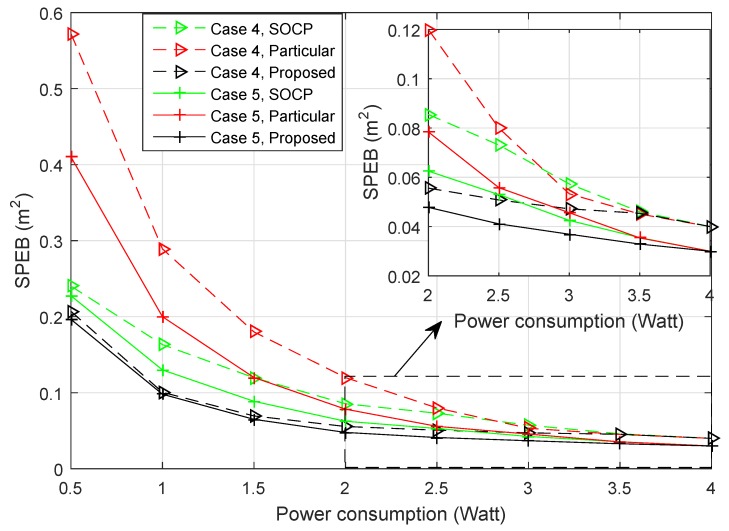
The average SPEB of agent nodes with respect to total power consumption of anchor nodes. SOCP, second-order cone program.

Figure 10 shows the average SPEB of agent nodes and the total power consumption of anchor nodes with respect to different anchor-specific power conservation levels *V*. Figure 11 shows the average SPEB of SOCP-based game strategies for particular agent nodes and the proposed strategies with respect to the total power consumption of anchor nodes. We can draw the following observations. First, for the proposed game power allocation strategies, different numbers of agent nodes have similar localization accuracy with the same total power consumption in Case 4 and Case 5. This is because the strategy depends on the estimation distribution. In different cases, the difference of estimation distributions is insignificant. For the SOCP-based and game strategies for particular agent nodes, the difference of localization accuracy and power consumption between Case 4 and Case 5 is obvious. This is because the more agent nodes are involved, the more strategies are obtained, then the power allocation strategies will be more reasonable for all agent nodes. Second, in both cases, the proposed power allocation strategies significantly outperform the SOCP-based and game strategies for particular agent nodes. With the same power conservation level V, not only the total power of proposed strategies is lower, but also the localization accuracy of the proposed strategies is much better than the game strategies for particular agent nodes. This means that the proposed game power allocation strategies are more energy-saving and utilize the power more efficiently. With the same power consumption, the localization accuracy of the proposed algorithm is better than the SOCP-based algorithm. This is because the SOCP-based strategies only depend on the position of a particular agent node. When all agent nodes in the service area are considered, the strategies may be unsuitable. At the same time, the performance of SOCP-based strategies outperforms the game strategies for particular agents. This is because the SOCP-based strategies have centralized computation for global optimal solutions; while in an exact potential game, the solutions are global or local optimal. Third, the cost of the proposed algorithm is that more agent nodes’ positions are considered rather than a particular one, so we need to obtain the distribution probabilities of agent nodes through prior information or extra estimation. To the best of our knowledge, when the total agent nodes are considered at the same time, the SOCP-based method may not solve such a problem effectively.

## 5. Conclusions

In this paper, we investigated power allocation strategies by considering the service area and the distribution of agent nodes in a WSN localization system. To obtain efficient power allocation strategies, a power management game is proposed where the utility function of anchor nodes is related to its own power consumption and the localization accuracy of all agent nodes in the service area. In addition, the proposed game is proven to be a continuous exact potential game after a potential function is constructed. Due to the continuous characteristic of the power management game, the end solution is achieved through a better response learning algorithm. Simulation results illustrate that when prior information is accessible, the proposed strategies outperform uniform strategies. When there is no prior information, the estimated distribution of agent nodes can be obtained first. Based on the estimated distribution, the proposed strategies outperform SOCP-based and game strategies for particular agent nodes. At the same time, changes in the different power conservation levels *V* result in a trade-off between localization accuracy and power consumption, which provides a guideline for operations in WSN localization systems.

## Figures and Tables

**Table 1:** Complexity of Algorithm 1.

No.	Step	Computation	Operation	Size	Cost
1	Step 4	$\cos\phi {}_{j}^{\prime }=\frac{\left(\varsigma_{n}-x_{j}\right)}{\sqrt{{\left(\varsigma_{n}-x_{j}\right)}^{2}+{\left(\eta_{n}-y_{j}\right)}^{2}}}$	3 sums, 2 products, 2 divisions, 1 square root	*N*	O(8N)
		$\sin\phi {}_{j}^{\prime }=\frac{\left(\eta_{n}-y_{j}\right)}{\sqrt{{\left(\varsigma_{n}-x_{j}\right)}^{2}+{\left(\eta_{n}-y_{j}\right)}^{2}}}$			
2	Step 4	$J_{r}\left(\phi \right)\overset{\Delta }{=}\left[\begin{array}{cc} \cos^{2}\phi & \cos\phi \sin\phi \\ \cos\phi \sin\phi & \sin^{2}\phi \end{array}\right]$	4 products	*N*	O(4N)
3	Step 4	$p_{j}\xi_{j}J_{r}\left(\phi_{j}^{\prime }\right)$	$5N_{b}$ products	*N*	$O(5NN_{b})$
		$\sum_{j\in N_{b}}p_{j}\xi_{j}J_{r}\left(\phi_{j}^{\prime }\right)$	$4N_{b}$ sums	*N*	$O(4NN_{b})$
4	Step 4	$tr[{\left(\sum_{j\in N_{b}}p_{j}\xi_{j}J_{r}\left(\phi_{j}^{\prime }\right)\right)}^{-1}]$	matrix inverse (2 × 2)	*N*	$O(2^{3}N)$
			2 sums	*N*	O(2N)
5	Step 4	$\hat{P}\left(z_{\left(x,y\right)};p\right)\approx$	2 products	*N*	O(2N)
		$\sum_{n=1}^{N}\mathrm{tr}\left\{{\left(\sum_{j\in N_{b}}p_{j}\xi_{j}J_{r}\left(\phi_{j}^{\prime }\right)\right)}^{-1}\right\}f\left(\varsigma_{n},\eta_{n}\right)\Delta \sigma_{n}$	(*N*-1) sums	1	O(N−1)
6	Step 5	$A_{k}^{*}\left(t\right)=\left[p_{k}\left(t\right),p_{k}^{{}^{\prime }}\left(t\right),p_{k}^{{}^{\prime \prime }}\left(t\right)\right]$	2 sums	1	O(2)
7	Step 5	$p_{k}\left(t+1\right)=\arg\max_{p_{k}\in A_{k}^{*}}u_{k}\left(p_{k},p_{-k}|t\right)$	sum (Nos. 3 to 5) *	≤2	$\leq 2[O\left(5NN_{b}\right)+O\left(4NN_{b}\right)+O\left(2^{3}N\right)+2O\left(2N\right)+O\left(N-1\right)]$

* sum (Nos. 3 to 5) means the summation operations from No. 3 to No. 5.
